# Unmet healthcare needs predict frailty onset in the middle-aged and older population in China: A prospective cohort analysis

**DOI:** 10.3389/fpubh.2023.1064846

**Published:** 2023-02-06

**Authors:** Jun Li, Di Wu, Haomiao Li, Jiangyun Chen

**Affiliations:** ^1^Wuhan Children's Hospital (Wuhan Maternal and Child Healthcare Hospital), Tongji Medical College, Huazhong University of Science and Technology, Wuhan, China; ^2^School of Political Science and Public Administration, Wuhan University, Wuhan, China; ^3^School of Health Management, Southern Medical University, Guangzhou, China

**Keywords:** healthy aging, unmet healthcare needs, frailty, China, middle-aged and older

## Abstract

**Objectives:**

Older populations have a relatively high prevalence of unmet healthcare needs, which can result in poor health status. Moreover, in the coming century, frailty is expected to become one of the most serious global public health challenges. However, there is a lack of clear evidence proving an association between unmet healthcare needs and frailty. This study aimed to assess whether unmet healthcare needs predict the onset of frailty in China.

**Methods:**

The association between frailty and unmet healthcare needs was explored by analyzing data from the China Health and Retirement Longitudinal Study (CHARLS) using random-effects logistic regression and Cox regression with time-varying exposure.

**Results:**

At baseline, 7,719 respondents were included in the analysis. Random-effects logistic regression shows that unmet outpatient healthcare needs were associated with increased risk of both contemporaneous (adjusted OR [aOR], 1.17; 95% CI, 1.02–1.35) and lagged (aOR, 1.24; 95% CI, 1.05–1.45) frailty, as were unmet inpatient needs (contemporaneous: aOR, 1.28; 95% CI, 1.00–1.64; lagged: aOR, 1.55; 95% CI, 1.17–2.06). For respondents not classified as frail at baseline (*n* = 5,392), Cox regression with time-varying exposure shows significant associations of both unmet outpatient needs (adjusted HR, 1.23; 95% CI, 1.05–1.44) and unmet inpatient needs (adjusted HR, 1.48; 95% CI, 1.11–1.99) with increased risk of developing frailty.

**Conclusions:**

Reducing unmet healthcare needs would be a valuable intervention to decrease frailty risk and promote healthy aging in middle-aged and older populations. It is urgent and essential that the equity and accessibility of the medical insurance and health delivery systems be strengthened.

## 1. Introduction

Unmet healthcare needs (UHN) is defined as the subjective perception of not receiving appropriate medical help when required. It captures misalignment between needs and provision and is commonly used as an indicator of access to healthcare and to precisely evaluate health system performance (1). Equitable access to healthcare according to need, regardless of demographic characteristics, ability to pay or social background is the core aim of universal health coverage (UHC) and thus UHC is undermined where there are UHN, as these can result in poorer health status, consequent risk of catastrophic health expenditures and increasing health inequalities (2, 3). China has moved to reduce UHN and promote health equity through introducing a series of reforms, such as the New Medical Reform in 2009 and increasing the level of health insurance compensation. However, UHN remain, potentially because some residents experience greater economic burden and traveling inconvenience in accessing healthcare and have less health literacy and trust in medical institutions or healthcare providers than others (3–5).

There is a higher prevalence of UHN in older populations. Population aging is leading to a growth in the number of older adults who are functionally or cognitively impaired, increasing their need for healthcare resources (3). However, they may also be at higher risk of being left unattended, economically unsupported or immobile. In China in particular, the current infrastructure and health system are not well-designed to meet the needs of the older population. This mismatch between need and resource access inevitably increases the number of older people with UHN. China has become an aging society and as this continues, the burden borne by family and public healthcare systems will still increase (6). Thus, the UHN of older populations are a major challenge for China's healthy aging strategy.

The core components of the healthy aging strategy in China are to establish a nationwide, affordable, annual health check system to facilitate early diagnosis and provide access to affordable treatments and to enable the elderly to live healthier, happier, longer and more productive lives (7). Thus, it positions strengthening equity in healthcare and improving health-related quality of life for older populations as the two main measures of healthy aging. UHN reduce health equity and quality of life, which has been linked to frailty. Frailty, a state of overall decline in physical, mental or cognitive functions ranging from mild to severe (8, 9), is an important cause of decreased quality of life and is associated with disease prevalence and severity (10). As numerous studies have shown a relationship between frailty and health, frailty management is seen as an important component of the healthy aging strategy.

Previous studies have shown that UHN may worsen health status and quality of life (11), leading to an increased risk of mortality (12), multimorbidity (13), depression (14, 15), dementia and mild cognitive impairment (16), which can interfere with the person's ability to perform instrumental activities of daily living (IADLs) (17). Therefore, it is possible that UHN are associated with the onset of frailty. However, clear evidence of such an association is so far lacking. In view of this, we conducted a longitudinal study to analyse whether UHN predict the onset of frailty in China with the aim of generating recommendations for the promotion of healthy aging and health equity in older populations.

## 2. Methods

### 2.1. Sample and data

Data for this study were obtained from the four waves of the China Health and Retirement Longitudinal Study (CHARLS) conducted in 2011, 2013, 2015, and 2018. Owing to its high quality and national representativeness, this micro-level database on middle-aged and older adults has been widely used for geriatrics-focused clinical, public health and sociological research in China. The 17,708 individual respondents included in the baseline 2011 survey were followed up once every 2 or 3 years; detailed descriptions of the sampling and variables measured are reported elsewhere (18). The sample inclusion criteria for the current study were: (1) responding and at least 45 years old in the 2011 wave; (2) investigated in at least one follow-up wave; (3) not missing values for the dependent (frailty) and independent (UHN) variables. The final sample comprised 7,719 respondents at baseline, 1,787 suffering from frailty and 5,392 not frail.

### 2.2. Variables

#### 2.2.1. Exposure: UHN

UHN were assessed by whether participants reported that either outpatient services or inpatient services did not meet their needs. Outpatient healthcare needs were considered unmet if respondents had been ill in the last month (at review) but had not visited a provider (including public hospitals, private hospitals, public health centers, clinics, or health worker's or doctor's practices) or been visited by a health worker or doctor for outpatient care. Inpatient healthcare needs were considered unmet if a doctor had indicated a need for inpatient care in the past year but the respondent had not been hospitalized.

#### 2.2.2. Outcome: Frailty

Frailty, the outcome variable of this study, was measured based on Rookwood's Cumulative Deficit Frailty Index (FI). The deficits used to calculate FI were selected according to the following inclusion criteria: (1) a minimum of 30 deficits; (2) associated with adverse health outcomes; (3) increase in prevalence with age at least into the tenth decade; (4) at last 1% prevalence in the population; (5) does not saturate (19). FI was calculated using 48 deficits, with binary variables coded as 0 or 1 and some ordered categorical variables coded as 0, 0.5 and 1. The details are presented in Supplementary material Page 1–2. Because chronic conditions are irreversible, a score of 1 in one wave was extended to all subsequent waves. FI was calculated by summing the number of deficits reported by the participant and dividing it by the total number of deficits they had responded on, generating an FI ranging from 0 to 1, with higher FI indicating more severe frailty. FI was then categorized using a defined cut-off point of 0.25 (i.e., robust or prefrail: < 0.25; frail: ≥0.25–1.00) (20, 21).

#### 2.2.3. Covariates

Respondents' demographic and economic characteristics were used as controlling covariates in the analysis. These comprised age, gender, marital status, Hukou status, education level, rural or urban residence, public health insurance coverage, current work status and household per capita consumption. The grouping details are presented in Table 1.

**Table 1:** Baseline characteristics.

	**Outpatient needs**			**Inpatient needs**		
	**Met**	**Unmet**	***P*** **-value**	**Met**	**Unmet**	***P*** **-value**
Number of participants	6,466	713		6,319	157	
Age	58.43 ± 10.50	58.73 ± 10.19	0.496	58.43 ± 10.53	57.78 ± 9.33	0.466
Gender			0.215			0.145
Male	3,088 (47.76%)	358 (50.21%)		3,009 (47.63%)	84 (53.50%)	
Female	3,377 (52.24%)	355 (49.79%)		3,309 (52.37%)	73 (46.50%)	
Marital status			0.011			0.677
Divorced or widowed	742 (13.34%)	60 (9.71%)		725 (13.36%)	17 (12.14%)	
Married	4,819 (86.66%)	558 (90.29%)		4,703 (86.64%)	123 (87.86%)	
Education levels^a^			0.107			0.003
Less than lower secondary	5,644 (87.33%)	642 (90.04%)		5,530 (87.56%)	123 (78.34%)	
Upper secondary and vocational training	678 (10.49%)	60 (8.42%)		651 (10.31%)	28 (17.83%)	
Tertiary	141 (2.18%)	11 (1.54%)		135 (2.14%)	6 (3.82%)	
Hukou status^b^			0.047			0.438
Agricultural	4,269 (76.81%)	480 (77.67%)		4,170 (76.87%)	103 (73.57%)	
Non-agricultural	1,265 (22.76%)	131 (21.20%)		1,231 (22.69%)	37 (26.43%)	
Other	24 (0.43%)	7 (1.13%)		24 (0.44%)	0 (0.00%)	
Rural/urban residence			0.052			0.023
Rural	3,755 (58.07%)	441 (61.85%)		3,654 (57.83%)	105 (66.88%)	
Urban	2,711 (41.93%)	272 (38.15%)		2,665 (42.17%)	52 (33.12%)	
Current work status			0.516			0.366
Not working	2,241 (35.23%)	239 (34.00%)		2,188 (35.20%)	60 (38.71%)	
Working	4,120 (64.77%)	464 (66.00%)		4,028 (64.80%)	95 (61.29%)	
Household per capita consumption^c^			0.072			0.710
Low	1,792 (32.95%)	188 (32.41%)		1,751 (32.96%)	46 (33.82%)	
Low to middle	1,508 (27.73%)	189 (32.59%)		1,468 (27.63%)	42 (30.88%)	
Middle	1,241 (22.82%)	116 (20.00%)		1,217 (22.91%)	26 (19.12%)	
High	898 (16.51%)	87 (15.00%)		877 (16.51%)	22 (16.18%)	
Public health insurance coverage^d^			0.686			0.878
Not covered	458 (8.28%)	48 (7.80%)		447 (8.28%)	11 (7.91%)	
Covered	5,075 (91.72%)	567 (92.20%)		4,954 (91.72%)	128 (92.09%)	

a Education levels were classified by a simplified version of the 1997 International Standard Classification of Education codes.

b Hukou status indicates the respondent's hukou place and is a special identifier in China. Hukou status affects many aspects of life in China such as buying a house, buying a car, children's school enrollment and other welfare.

c Household per capita consumption was calculated by dividing total household consumption by the number of people in the household, where total household consumption was an aggregate of food consumption in the last week, non-food consumption in the past 30 days and other non-food consumption in the past year. The household per capita consumption values for the different survey waves were adjusted using the Consumer Price Index and then divided into quartiles.

d Public health insurance includes Urban Employee Medical Insurance, Urban Resident Medical Insurance, New Cooperative Medical Insurance, Urban and Rural Resident Medical Insurance, Government Medical Insurance, Medical Aid or other government insurance plan.

### 2.3. Statistical analysis

First, the baseline characteristics of the sample were evaluated by calculating the mean and standard deviation for continuous variables (age) and frequency and percentage for categorical variables. Statistical differences for age were tested by Kruskal-Wallis one-way analysis as it was not normally distributed, and differences for categorical variables were tested using and Chi-square tests.

Second, random-effects logistic regression was employed using a panel data approach to examine the association between UHN and frailty. Only data from 2011 to 2015 were used because UHN were not measured in the 2018 wave. Both contemporaneous (2011–2015) and lagging (2013–2018) frailty status were set as dependent variables due to the potential for a time-lagged impact of UHN on frailty. Odds ratios (OR) were calculated with a 95% confidence interval.

Third, the relative risk of frailty was calculated by including respondents who were non-frail at baseline and employing Cox regression to assess the predictive effect, with survey waves as the timescale and those who remained non-frail treated as censored data. UHN were identified based on 2011–2015 data and frailty status on 2013–2018 data. In addition, as economic status, health-seeking behavior and medical insurance compensation level may change over time, affecting whether healthcare needs are met, UHN were treated as a time-varying exposure to avoid immortal time bias (22). Hazard ratios (HR) were calculated with a 95% confidence interval.

Multiple imputation using all the covariates was performed before conducting the above regressions to avoid reductions in statistical test performance and bias caused by the direct exclusion of missing values. Five replications and chained equations were used to generate five sets of databases and calculate a pooled regression coefficient (23, 24). The details of missing values were shown in Supplementary Table S1.

The validity of the results was assessed using three sensitivity analyses. First, Cox regression with time-varying exposure was performed on data without imputation. Second, respondents without any loss-up (2011–2018) were included in the Cox analysis. Third, the methodology developed by VanderWeele and Ding was used to calculate E-values, an alternative approach for detecting unmeasured confounding (25).

In addition, as China's rural and urban areas differ significantly in terms of economic level, health insurance compensation level, proportion of older people, family structures and healthcare resources, we explored the impact of UHN on frailty for rural and urban participants separately.

## 3. Results

**Figure 1:**
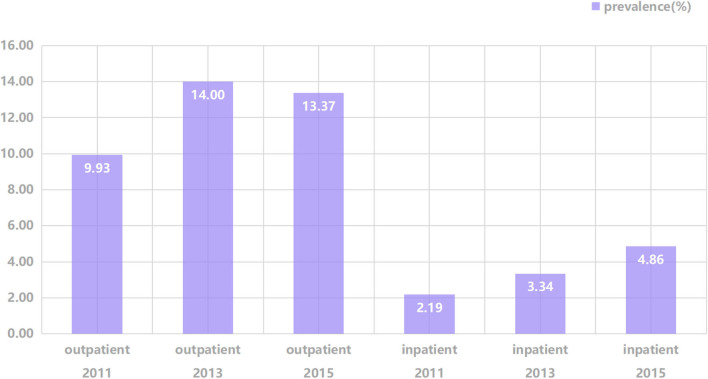
Prevalence of unmet healthcare needs across survey waves.

Table 1 describes the baseline characteristics of participants with and without unmet outpatient and inpatient needs. The met and unmet groups generally have similar characteristics, except for marital status and Hukou status in the outpatient analysis and education levels and residence in the inpatient analysis. The ratio of inpatient needs is significantly higher for rural residents. The prevalence of UHN is shown in Figure 1. For unmet outpatient needs, it was 9.93% in 2011 and increased to 14% in 2013 then decreased to 13.37% in 2015. The prevalence of unmet inpatient needs was 2.19% in 2011, increasing to 3.34% in 2013 and then 4.86% in 2015.

Random-effects logistic regression shows that unmet outpatient healthcare needs were associated with increased risk of both contemporaneous (adjusted OR, 1.17; 95% CI, 1.02–1.35) and lagged (adjusted OR, 1.24; 95% CI, 1.05–1.45) frailty, as were unmet inpatient needs (contemporaneous: adjusted OR, 1.28; 95% CI, 1.00–1.64; lagged: adjusted OR, 1.55; 95% CI, 1.17–2.06) (Table 2).

**Table 2:** Association between UHN and frailty based on random-effects logistic regression.

	**Outpatient services**				**Inpatient services**			
	**Not lagged**		**Lagged**		**Not lagged**		**Lagged**	
	**OR (95%CI)**	***P*** **-value**	**OR (95%CI)**	***P*** **-value**	**OR (95%CI)**	***P*** **-value**	**OR (95%CI)**	***P*** **-value**
Unmet healthcare needs (ref. Met)	1.17 (1.02, 1.35)	0.021	1.24 (1.05, 1.45)	0.009	1.28 (1.00, 1.64)	0.046	1.55 (1.17, 2.06)	0.002
Age	1.07 (1.06, 1.08)	< 0.001	1.05 (1.04, 1.05)	< 0.001	1.07 (1.07, 1.08)	< 0.001	1.05 (1.04, 1.06)	< 0.001
Gender (ref. Male)	1.93 (1.72, 2.17)	< 0.001	1.85 (1.62, 2.11)	< 0.001	1.95 (1.72, 2.2)	< 0.001	1.90 (1.66, 2.18)	< 0.001
**Education level (ref. less than lower secondary)**								
Upper secondary and vocational training	1.03 (0.83, 1.27)	0.793	0.56 (0.44, 0.71)	< 0.001	1.00 (0.81, 1.25)	0.978	0.54 (0.42, 0.69)	< 0.001
Tertiary	1.42 (0.93, 2.17)	0.101	0.72 (0.44, 1.18)	0.195	1.35 (0.87, 2.09)	0.174	0.71 (0.43, 1.17)	0.176
Marital status (ref. Divorced or widowed)	0.83 (0.72, 0.97)	0.019	0.66 (0.56, 0.79)	< 0.001	0.83 (0.71, 0.97)	0.023	0.72 (0.6, 0.87)	< 0.001
Rural/urban residence (ref. Rural)	0.90 (0.80, 1.01)	0.075	0.80 (0.7, 0.92)	0.002	0.89 (0.79, 1.01)	0.069	0.80 (0.69, 0.92)	0.001
Current work status (ref. Not working)	0.94 (0.85, 1.03)	0.177	1.00 (0.9, 1.12)	0.990	0.91 (0.82, 1.01)	0.090	0.98 (0.87, 1.1)	0.758
**Household per capita consumption group (ref. low)**								
Low to middle	1.00 (0.88, 1.13)	0.973	0.90 (0.78, 1.04)	0.150	0.97 (0.85, 1.12)	0.713	0.87 (0.75, 1.02)	0.079
Middle	0.93 (0.82, 1.06)	0.256	1.03 (0.89, 1.19)	0.674	0.93 (0.81, 1.07)	0.313	1.03 (0.88, 1.20)	0.718
High	0.93 (0.81, 1.06)	0.263	1.14 (0.99, 1.33)	0.076	0.91 (0.8, 1.05)	0.214	1.12 (0.96, 1.32)	0.148
Hukou status (ref. Agricultural)	0.41 (0.36, 0.47)	< 0.001	1.16 (1.00, 1.34)	0.043	0.42 (0.37, 0.49)	< 0.001	1.10 (0.95, 1.27)	0.223
Public health insurance coverage (ref. Not covered)	0.94 (0.79, 1.11)	0.475	0.78 (0.65, 0.95)	0.012	1.01 (0.84, 1.21)	0.906	0.77 (0.63, 0.93)	0.008

UHN, unmet healthcare needs; OR, odds ratio; CI, Confidence Interval.

For respondents not classified as frail at baseline (baseline characteristics shown in Supplementary Table S2), Cox regression with time-varying exposure (Table 3) shows significant associations of both unmet outpatient needs (adjusted HR, 1.23; 95% CI, 1.05–1.44) and unmet inpatient needs (adjusted HR, 1.48; 95% CI, 1.11–1.99) with increased risk of developing frailty.

**Table 3:** Association between UHN and frailty based on Cox regression with time-varying exposure.

	**Outpatient services**		**Inpatient services**	
	**Odds ratio** **(95%CI)**	***P*** **-value**	**Odds ratio** **(95%CI)**	***P*-value**
Unmet healthcare needs (ref. Met)	1.23 (1.05, 1.44)	0.010	1.48 (1.11, 1.99)	0.008
Age	1.02 (1.01, 1.02)	< 0.001	1.02 (1.01, 1.02)	< 0.001
Gender (ref. Male)	1.34 (1.21, 1.48)	< 0.001	1.34 (1.2, 1.48)	< 0.001
**Education level (ref. Less than lower secondary)**				
Upper secondary and vocational training	0.88 (0.73, 1.05)	0.161	0.88 (0.73, 1.05)	0.164
Tertiary	1.21 (0.83, 1.76)	0.331	1.19 (0.82, 1.74)	0.358
Marital status (ref. Divorced or widowed)	0.73 (0.63, 0.84)	< 0.001	0.73 (0.63, 0.84)	< 0.001
Rural/urban residence (ref. Rural)	0.95 (0.85, 1.06)	0.332	0.95 (0.85, 1.06)	0.356
Current work status (ref. Not working)	0.98 (0.88, 1.09)	0.735	0.99 (0.88, 1.1)	0.790
**Household per capita consumption group (ref. Low)**				
Low to middle	0.88 (0.77, 1.02)	0.082	0.88 (0.77, 1.02)	0.083
Middle	0.93 (0.81, 1.07)	0.341	0.93 (0.81, 1.08)	0.343
High	0.85 (0.73, 0.99)	0.043	0.85 (0.73, 1)	0.044
**Hukou status (ref. Agricultural)**				
Non-agricultural	1.12 (0.99, 1.28)	0.072	1.12 (0.99, 1.27)	0.083
Other	0.82 (0.47, 1.44)	0.491	0.83 (0.47, 1.45)	0.509
Public health insurance coverage (ref. Not covered)	0.71 (0.60, 0.83)	< 0.001	0.71 (0.6, 0.83)	< 0.001

UHN, unmet healthcare needs; HR, hazard ratio; CI, Confidence Interval.

The results of the three sensitivity analyses are presented in Table 4, Supplementary Tables S3, S4. First, the results of using non-imputed data to repeat the Cox regression were consistent with the main analysis. Second, results similar to the main analysis were obtained when using data of respondents without loss-up, except that the effect of unmet outpatient needs was not significant at the 95% level (P = 0.087). Third, the E-values were greater than the estimated confounders for frailty, which indicates that a potential unmeasured confounder is unlikely to have a considerably greater effect on frailty than the known risk factors.

**Table 4:** Sensitivity analysis^*^.

	**Unmet healthcare needs (ref. Met)**	
	**HR (95%CI)**	***P*** **-value**
**Without imputation**		
Outpatient	1.26 (1.04, 1.53)	0.016
Inpatient	1.47 (1.03, 2.11)	0.036
**Without loss up**		
Outpatient	1.19 (0.98, 1.45)	0.087
Inpatient	1.58 (1.13, 2.20)	0.007

HR, hazard ratio; CI, Confidence Interval.

* Cox regression with time-varying exposure were used to calculate relative risks. The adjusted confounders includes age, gender, marital status, Hukou status, education level, rural or urban residence, public health insurance coverage, current work status and household per capita consumption.

We compared how UHN affect frailty for those in urban and rural areas (Table 5). Notably, a significant increase in frailty risk in those with both unmet outpatient and inpatient needs (vs. those with met needs) was seen only in respondents in rural areas (outpatient: adjusted HR, 1.23; 95% CI, 1.01–1.50; inpatient: adjusted HR, 1.47; 95% CI, 1.04–2.08), not those in urban areas.

**Table 5:** Subgroup analysis of urban/rural residence^*^.

	**Unmet healthcare needs (ref. Met)**	
	**HR(95%CI)**	***P*** **-value**
**Urban**		
Outpatient	1.24 (0.95, 1.60)	0.110
Inpatient	1.49 (0.86, 2.56)	0.152
**Rural**		
Outpatient	1.23 (1.01, 1.50)	0.037
Inpatient	1.47 (1.04, 2.08)	0.028

HR, hazard ratio; CI, Confidence Interval.

* Cox regression with time-varying exposure were used to calculate relative risks. The adjusted confounders includes age, gender, marital status, Hukou status, education level, rural or urban residence, public health insurance coverage, current work status and household per capita consumption.

We further analyzed reasons for UHN. For unmet outpatient needs (Supplementary Figure S1), “illness is not serious” was the main cause (2011, 48.93; 2013, 57.97, 2015, 51.18%). For unmet inpatient needs (Supplementary Figure S2), ‘not having enough money' was the main cause (2011, 61.08; 2013, 61.29, 2015, 57.48%).

## 4. Discussion

To the best of our knowledge, this is the first study to assess the effect of UHN on the development of frailty. This study uses nationally representative data and cohort analysis to show that both unmet outpatient and inpatient healthcare needs increase frailty risk in China's middle-aged and older population. Nevertheless, this effect was pronounced only in rural China.

Given the multiple adverse outcomes associated with frailty, such as falls and fractures, hospitalisations, reduced quality of life, complications and early mortality (26), it will undoubtedly be among the most serious global public health challenges in the coming century (10). China has the world's largest older population, with the Seventh National Census showing 264 million people to be 60 years old and over. The rapid expansion of the aging population has brought about a concomitant rise in the frequency of frailty, placing increased pressure on healthcare systems (27, 28). As frailty increases the need for medical services, the unmet need for services also increases. Previous studies have shown that frailty increases UHN (29). However, UHN also increase the risk of frailty, which could be caused by several possible mechanisms. First, UHN in older adults have been found to be associated with deteriorating physical health. UHN usually brings about delay of disease treatment, the miss of the best treatment period of the disease, and even the subsequent aggravation of diseases (30, 31). Frailty itself is also a risk factor of disease aggravation, and UHN inevitably brings about an increased risk of frailty. Second, UHN have also been found to be associated with worse psychological health. UHN often lead to deteriorating health, which could affect the quality of life and lead to more depressive symptoms in several ways like adding chronic pain, limiting the ability to move and carry out valued activities, participating in social activities, or even taking care of themselves and their basic needs. Witnessing their deteriorating health might also add to feeling more negative emotions and helplessness about their situation and the future (32). In addition, frailty often coincides with chronic disease, and even multimorbidity. For older populations suffering chronic diseases, UHN often means insufficient or inadequate management, which could increase risk of co-occurence of chronic conditions and frailty (33, 34). These suggest that reducing UHN is crucial for delaying the onset of frailty and consequently promoting healthy aging.

The Chinese government has made tremendous efforts to reduce UHN by improving the accessibility of medical services and providing affordable and equitable healthcare for all residents. These efforts center around expanding healthcare insurance coverage, increasing the accessibility of basic public health services for all, establishing a national essential medicines system, improving basic healthcare delivery within the primary care system and reforming public hospitals (35, 36). However, although years of medical service system and medical insurance reform have positively impacted health service provision to residents, we found that the incidence of UHN, especially for inpatient services, has increased. This may be attributable to an increase in absolute demand as the population ages. We analyzed the reasons for not having their healthcare needs met and found that the perception that the “illness is not serious” was the main cause for unmet outpatient needs, followed by “not having enough money” and that “not having enough money” was the main cause of unmet inpatient healthcare needs. Firstly, these findings indicate that the health literacy and health awareness of older residents need to be improved. Secondly, as older people progressively become less able to work, their ability to pay for the use of medical services also decreases. Therefore, it is necessary to intervene to increase their medical security.

Notably, the impact of UHN on frailty is more significant in rural than in urban areas of China. A likely cause of this is the lower income level of rural older populations, which arises because, while the urban population has retirement wages, the rural population do not and suffer an irreversible decline in income as ability to work decreases (37, 38). Moreover, the ongoing population outflow from rural areas can weaken family cohesion and economic support to the elderly from their children may be lacking (39, 40). In addition, rural residents generally have less health literacy and health awareness than urban residents (41). All of these factors not only increase UHN in rural areas but cause worse overall health in older populations in rural than urban areas, making the onset of frailty more sensitive to the effects of UHN in rural China.

In summary, this study identifies a targeted means of frailty-reduction: reducing UHN. This reduction could be achieved by further improving the medical security system, strengthening social solidarity and family function and increasing medical service utilization. Especially in rural areas, we need more policy actions to gradually establish a more equitable long-term care mechanism and ensure the active supply of medical services, as this will reduce UHN and thus reduce the occurrence of frailty.

This study has several limitations. First, the use of self-report data will have introduced recall bias. For example, self-report of the deficits used to calculate the FI may not be accurate, affecting the accuracy of the frailty level estimation. Second, respondents who became frail but died before 2018 have not been included in our analysis. Given the association of frailty with mortality, excluding them may have introduced survival bias. Third, the FI amalgamates numerous indicators. Observations for many indicators were missing from individual participants' interview data, which led to sample loss. Nevertheless, we applied a cohort design and conducted several sensitivity analyses to overcome and check for this bias. Fourth, the prevalence of and reasons for UHN were not measured in 2018, making the results less informative about the current situation in China than they would be with more recent data. Finally, the reference group in this study was respondents without UHN, which included both those who were ill but receiving medical services and those who were not ill. We could not draw any conclusions about whether there was an increase in frailty risk for those with a given serious illness who had their healthcare needs unmet. However, based on our analysis, this study clearly indicates that UHN are an important predictive factor for frailty onset.

## 5. Conclusions

This study clearly demonstrated that UHN put middle-aged and older populations in China, especially in rural areas, at greater risk of becoming frail. Therefore, reducing UHN would be a valuable intervention to promote healthy aging in middle-aged and older populations. As population aging becomes an ever-greater challenge, it is urgent and essential to strengthen the equity and accessibility of the medical insurance and health delivery systems.

## Data availability statement

Publicly available datasets were analyzed in this study. This data can be found here: All the original data could be obtained from the official website of CHARLS (http://charls.pku.edu.cn/).

## Author contributions

JL, JC, and HL designed and conducted the research. JL, DW, and JC analyzed the data and interpreted the results. JL, DW, and HL prepared the first manuscript and had primary responsibility for the final content. All authors critically reviewed, interpreted the results, and approved the final manuscript.
