# Corrigendum: Unmet healthcare needs predict frailty onset in the middle-aged and older population in China: A prospective cohort analysis

**DOI:** 10.3389/fpubh.2023.1185209

**Published:** 2023-03-24

**Authors:** Jun Li, Di Wu, Haomiao Li, Jiangyun Chen

**Affiliations:** ^1^Wuhan Children's Hospital (Wuhan Maternal and Child Healthcare Hospital), Tongji Medical College, Huazhong University of Science and Technology, Wuhan, China; ^2^School of Political Science and Public Administration, Wuhan University, Wuhan, China; ^3^School of Health Management, Southern Medical University, Guangzhou, China

**Keywords:** healthy aging, unmet healthcare needs, frailty, China, middle-aged and older

In the published article, there was an error in affiliation 1. Instead of “Tongji Medical College, Wuhan Children's Hospital (Wuhan Maternal and Child Healthcare Hospital), Huazhong University of Science and Technology, Wuhan, China,” the affiliation should be “Wuhan Children's Hospital (Wuhan Maternal and Child Healthcare Hospital), Tongji Medical College, Huazhong University of Science and Technology, Wuhan, China.”

The authors apologize for this error and state that this does not change the scientific conclusions of the article in any way. The original article has been updated.

