# Supplementary material for: Unmet healthcare needs predict frailty onset in the middle-aged and older population in China: A prospective cohort analysis
Source: Front Public Health. 2023 Feb 6;11:1064846. doi: 10.3389/fpubh.2023.1064846 (PMC9939901; doi:10.3389/fpubh.2023.1064846)
Supplement: Supplementary file 1 [file Data_Sheet_1.pdf]

Supplementary Material

1 Deficits to calculate frailty index

Frailty index was calculated by summing the number of deficits reported by the participant and dividing it by the total number of deficits they had responded on.

The selected deficits include physical limitations, depressive symptoms, cognition impairments, co-morbidities, and others.

Physical limitations include 19 items, with “1” indicating some difficulty with the activity or could not do the activity, and “0” indicating that the respondent did not have any problems with the activity. The details are as follows:

Have difficulty with: walking 100 Metres; walking 1 Km; controlling urination and defecation; getting up from chair after sitting for long periods; climbing several flights of stairs without resting; stooping, kneeling,or crouching; reaching or extending arms above shoulder level; lifting or carrying weights over 10 Jin (5kg); picking up a small coin from a table; dressing; bathing or showering; eating; getting out of bed and walking; using the toilet, including getting up or down; managing money; taking medications; shopping for groceries; preparing a hot meal; cleaning house.

Depressive symptoms include 10 items:

Felt depressed much of the time during the past week.	0=Rarely or none of the time; 0.5= Some or a little of the time/ Occasionally or a moderate amount of the time; 1= Most or all of the time.
Felt sleep was restless.	
Was not happy most of the time.	
Felt lonely.	
Bothered by little things.	
Could not get going.	
Had trouble keeping mind on what is doing.	
Felt fearful.	
Felt everything they did during the past week was an effort.	0=Most or all of the time; 0.5=Some or a little of the [illegible]
Felt hopeful about the future.	

Co-morbidities include 14 chronic conditions, with “1” indicating “ever diagnosed” and “0” indicating “never diagnosed”. The chronic conditions include: hypertension, diabetes, lung disease, heart problem, stroke, psych problem, arthritis, dyslipidemia, liver disease, kidney disease, stomach/digestive disease, asthma, memory problem, cancer.

Cognition impairments were measured by whether the respondents could answer correct day of the month given; correct month given; correct year given; correct day given. 0=can correctly answer; 1=cannot correctly answer.

Self reported health was also treated as one of the deficits. 0=very good/ good; 0.5=fair; 1= poor/ very poor.

The frailty index was calculated by summing the number of deficits reported by the respondents and dividing by the total number of possible (answered) deficits. For the chronic disease variables (e.g., hypertension), a score of 1 in one wave was allocated in all subsequent waves as the conditions were irreversible. Then, a frailty index with a potential range from 0 to 1 was generated, with a higher score indicating more serious frailty.

2 Supplementary Figures and Tables

2.1 Supplementary Figures

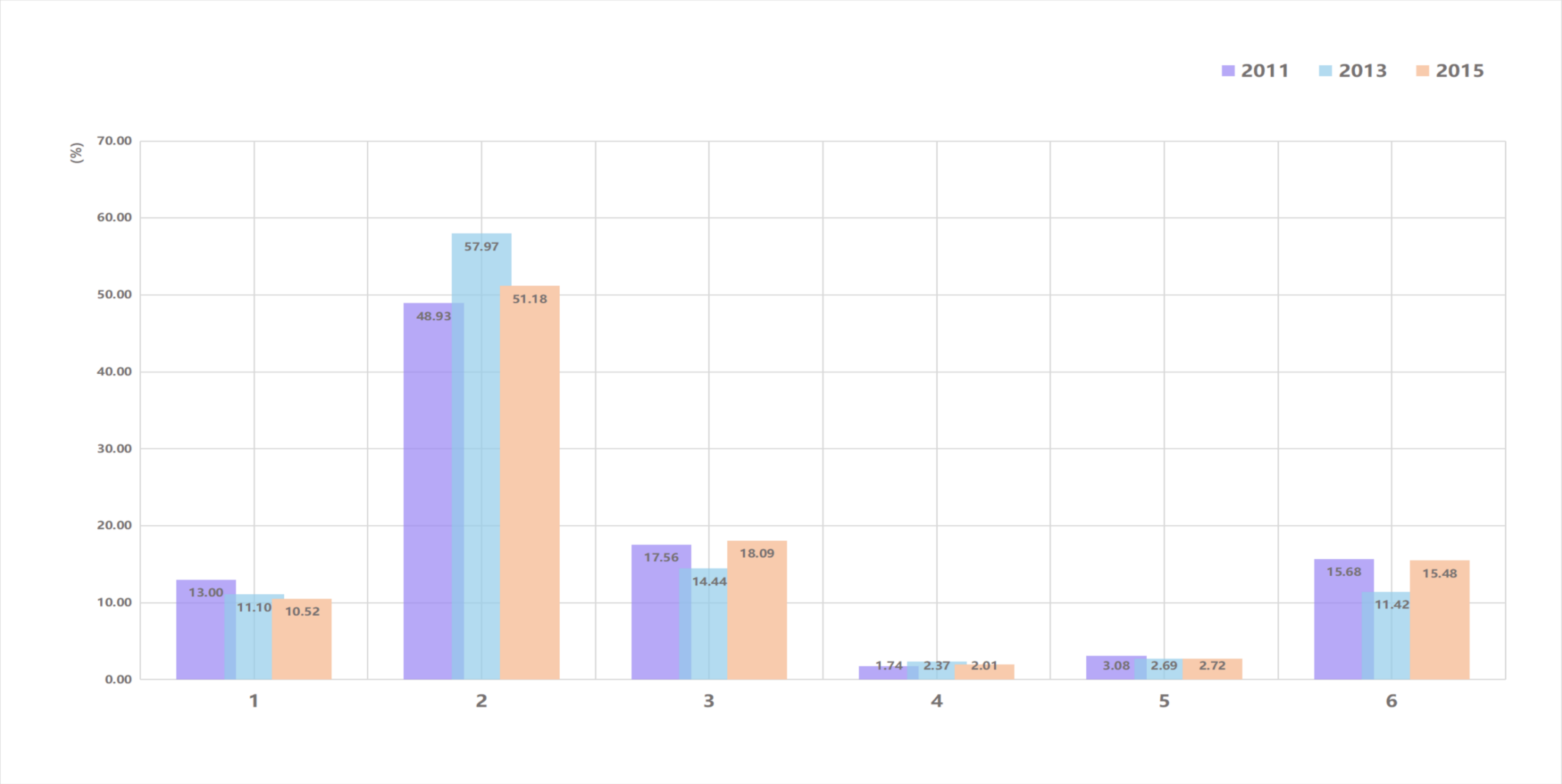

Supplementary Figure 1. Reasons for unmet outpatient needs. (1, Already Under Treatment; 2, Illness is not serious; 3, Not having enough money; 4, No time; 5, Inconvenient traffic; 6, other reasons.)

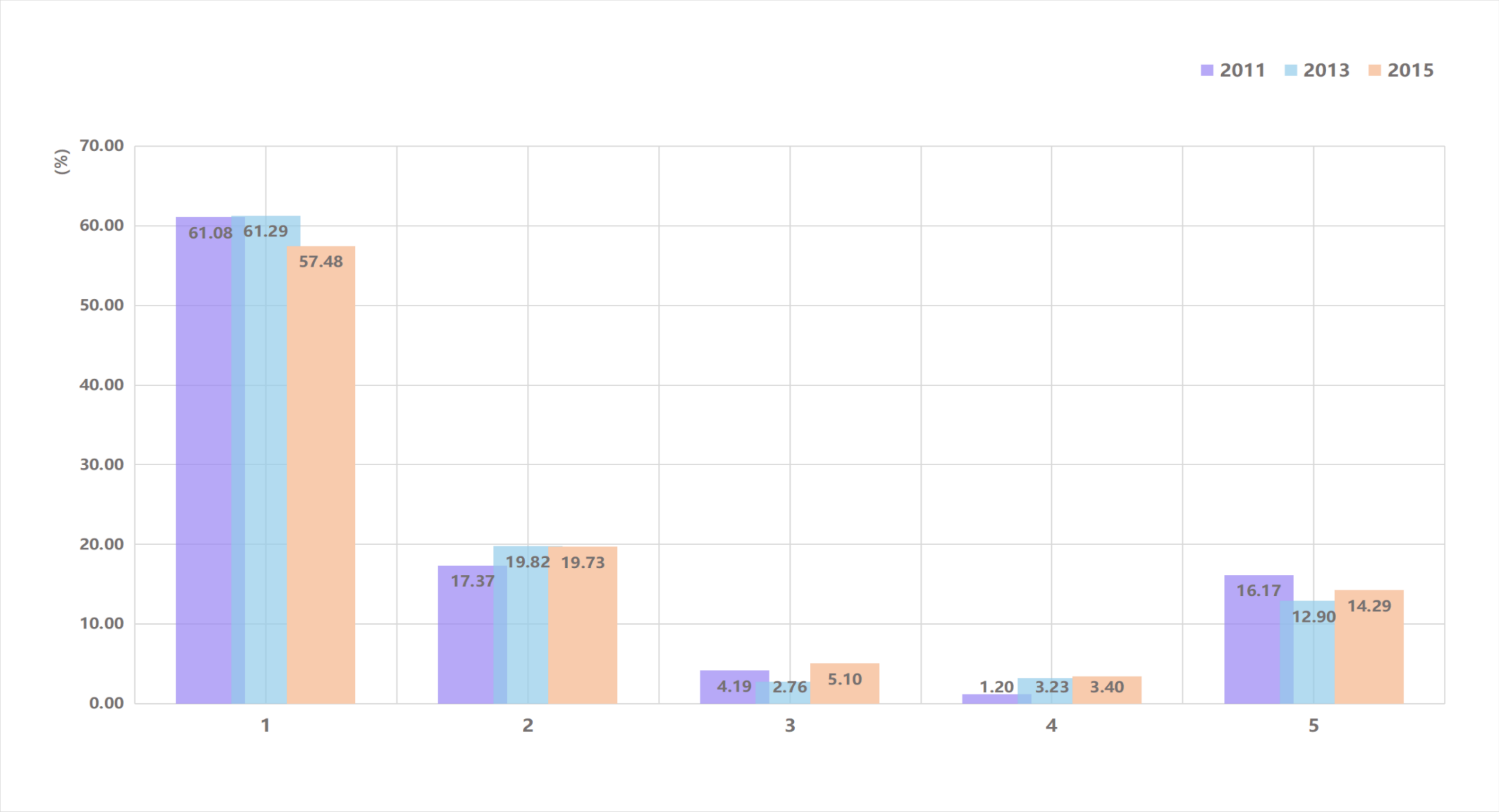

Supplementary Figure S2. Reasons for unmet inpatient needs. (1, Not having enough money; 2, Not willing to go to the hospital; 3, Poor Quality; 4, disease too serious; 5, other reasons.)

2.2 Supplementary Tables

Supplementary Table S1. Description of covariates before and after imputation*

Variable name	Variable type	Missing values	Mean	
			before imputation	after imputation
Age	continuous	2399	59.79	59.70
Gender	binary	2	1.52	1.52
Education levels	ordered-categorical	6	1.14	1.14
Marital status	binary	2305	0.86	0.86
Residence	binary	0	1.40	1.40
Hukou status	ordered-categorical	2568	1.24	1.23
Public health insurance coverage	binary	2567	0.92	0.92
Current work status	binary	1903	0.65	0.65
Household per capita consumption	ordered-categorical	5109	2.50	2.50

* The imputing method was from the posterior predictive distribution (ppd).

Supplementary Table S2. Baseline characteristics of baseline robust respondents

	Outpatient needs			Inpatient needs		
	met	unmet	P-value	met	unmet	P-value
Number of participants	4880	512		4773	112	
Age	57.08 ± 9.79	57.93 ± 10.13	0.070	57.10 ± 9.82	56.06 ± 8.51	0.277
Gender			0.298			0.123
Male	2436 (49.93%)	268 (52.34%)		2375 (49.77%)	64 (57.14%)	
Female	2443 (50.07%)	244 (47.66%)		2397 (50.23%)	48 (42.86%)	
Marital status			0.213			0.216
Divorced or widowed	504 (11.11%)	46 (9.27%)		496 (11.19%)	8 (7.41%)	
Married	4032 (88.89%)	450 (90.73%)		3937 (88.81%)	100 (92.59%)	
Education levels[a]			0.263			0.016
Less than lower secondary	4188 (85.87%)	453 (88.48%)		4107 (86.10%)	86 (76.79%)	
Upper secondary & vocational training	580 (11.89%)	49 (9.57%)		559 (11.72%)	21 (18.75%)	
Tertiary	109 (2.23%)	10 (1.95%)		104 (2.18%)	5 (4.46%)	
Hukou status[b]			0.019			0.248
Agricultural	3412 (75.25%)	378 (76.21%)		3340 (75.38%)	75 (69.44%)	
Non-agricultural	1101 (24.28%)	111 (22.38%)		1070 (24.15%)	33 (30.56%)	
Other	21 (0.46%)	7 (1.41%)		21 (0.47%)	0 (0.00%)	
Rural/urban residence			0.095			0.063
Rural	2806 (57.50%)	314 (61.33%)		2734 (57.28%)	74 (66.07%)	

Urban	2074 (42.50%)	198 (38.67%)		2039 (42.72%)	38 (33.93%)	
Current work status			0.552			0.085
Not working	1664 (34.65%)	169 (33.33%)		1619 (34.48%)	47 (42.34%)	
Working	3138 (65.35%)	338 (66.67%)		3077 (65.52%)	64 (57.66%)	
Household per capita consumption[c]			0.367			0.926
Low	1324 (32.17%)	133 (31.74%)		1294 (32.15%)	31 (32.63%)	
Low to middle	1150 (27.95%)	133 (31.74%)		1122 (27.88%)	29 (30.53%)	
Middle	941 (22.87%)	90 (21.48%)		923 (22.93%)	20 (21.05%)	
High	700 (17.01%)	63 (15.04%)		686 (17.04%)	15 (15.79%)	
Public health insurance coverage[d]			0.061			0.989
Not covered	375 (8.31%)	29 (5.88%)		366 (8.30%)	9 (8.33%)	
Covered	4140 (91.69%)	464 (94.12%)		4046 (91.70%)	99 (91.67%)	

[a] Education levels were classified by a simplified version of the 1997 International Standard Classification of Education codes.

[b] Hukou status indicates the respondent's hukou place and is a special identifier in China. Hukou status affects many aspects of life in China such as buying a house, buying a car, children's school enrollment and other welfare.

[c] Household per capita consumption was calculated by dividing total household consumption by the number of people in the household, where total household consumption was an aggregate of food consumption in the last week, non-food consumption in the past 30 days and other non-food consumption in the past year. The household per capita consumption values for the different survey waves were adjusted using the Consumer Price Index and then divided into quartiles.

[d] Public health insurance includes Urban Employee Medical Insurance, Urban Resident Medical Insurance, New Cooperative Medical Insurance, Urban and Rural Resident Medical Insurance, Government Medical Insurance, Medical Aid or other government insurance plan.

Supplementary Table S3. E-value related to Table 2

Random-effects logistic regression	Outpatient services				Inpatient services			
	Not lagged		Lagged		Not lagged		Lagged	
	OR (95%CI)	P value	OR (95%CI)	P value	OR (95%CI)	P value	OR (95%CI)	P value
Unmet healthcare needs (ref. Met)	1.17(1.02, 1.35)	0.021	1.24(1.05, 1.45)	0.009	1.28(1, 1.64)	0.046	1.55(1.17, 2.06)	0.002
E-value	1.38		1.47		1.52		1.8	

OR, odds ratio; CI, Confidence Interval.

Supplementary Table S4. E-value related to Table 3

cox regression	outpatient services		inpatient services	
	HR(95%CI)	P value	HR(95%CI)	P value
Unmet healthcare needs (ref. Met)	1.23(1.05, 1.44)	0.010	1.48(1.11, 1.99)	0.008
E-value	1.58		1.95	

HR, hazard ratio; CI, Confidence Interval.
